# Failure rate and treatment plan options of dental implants in patients with fibro-osseous lesions: a systematic review

**DOI:** 10.1186/s40902-026-00519-7

**Published:** 2026-07-14

**Authors:** Fazele Atarbashi-Moghadam, Amirali Farahani Afsaryeh, Saede Atarbashi-Moghadam, Amir-Ali Yousefi-Koma, Ali Azadi, Mahdi Kadkhodazadeh

**Affiliations:** 1 Shahid Beheshti University of Medical Sciences, Tehran, Islamic Republic of Iran; 2 Department of Periodontics, Dentistry, Shahid Beheshti University of Medical Sciences, Tehran, Iran; 3 Dental Research Center, Research Institute for Dental Sciences, Shahid Beheshti University of Medical Sciences, Tehran, Iran; 4 Department of Oral Pathology, School of Dentistry, Shahid Beheshti University of Medical Sciences, Tehran, Iran; 5 Dentofacial Deformities Research Center, Research Institute for Dental Sciences, Shahid Beheshti University of Medical Sciences, Tehran, Iran

**Keywords:** Fibrous Dysplasia of Bone, Cemento Ossifying Fibroma, Cemento-osseous Dysplasia, Dental Implants

## Abstract

**Background:**

Dental implant placement requires prerequisites, such as the quality and quantity of the available bone. Oral and maxillofacial fibro-osseous lesions (FOLs) are a group of intraosseous benign lesions, consisting of cemento-osseous dysplasia (COD), fibrous dysplasia (FD), and cemento-ossifying fibroma (COF), characterized by progressive replacement of normal bone tissue by fibrous connective tissue comprising varying amounts of mineralized substances, and consequently, the quality of the bone tissue. The aim of this study is to provide information for clinicians on the risks of implant insertion into these types of lesions.

**Main text:**

The ISI Web of Science, PubMed, and Scopus databases were searched based on the following PICO: The PICO question of the present study is as follows: What are the failure rates and treatment plan options (O) of implant placement (I) in patients with a fibro-osseous lesion who need an implant treatment at the site of the lesion (P)? Of the 301 identified studies, 39 eligible studies (16 on COD, 14 on FD, and nine on COF) were included in the present systematic review. Overall, 40 and 56 implants were placed in healthy areas near the lesions, in the grafted tissues placed in the location of the removed lesions, or within the COD and FD lesions, respectively. All of the 29 implants in the group of COF lesions were placed in the healed or grafted tissue, replacing the resected COF-affected areas. The failure rate of implants placed in the studies investigating COD, FD, and COF lesions were 22.50%, 7.14%, and 10.34%, respectively. Treatment options for implant placement in COD and FD lesions were reported as removing the lesion and waiting for the bone to be healed (with or without grafting), placing implant in the healthy bone near the lesion, or placing the implant embedded in the lesion. However, all of COF lesions were removed (mostly with resection) and dental implants were placed in the grafted tissue.

**Conclusion:**

Considering the limitations of the present study, it can be concluded that it would be more appropriate to avoid placing dental implants into the COD and FD lesions and inserting the implants in the surrounding healthy bone, healed, or grafted bone tissues are preferred. However, due to the neoplastic nature of COF removing the lesions prior to the implant insertion is crucial.

## Introduction

Dental implant placement requires prerequisites, such as available bone. Clinicians overcome bone deficiency through different techniques, including guided bone regeneration (GBR) [1]. However, the quality of this available bone is also critical. The success of dental implant treatment varies across different bone types [2, 3]. Thus, various protocols have been presented to increase the dental implant success rate in different types of bone [2]. In some situations, the jaw bones are affected by osseous lesions that have an impact on dental treatments, including oral rehabilitation with dental implants. Since these conditions are not seen routinely, deciding on a treatment plan for these patients can be challenging for dentists [4, 5].

Oral and maxillofacial fibro-osseous lesions (FOLs) are a group of intraosseous benign lesions characterized by progressive replacement of normal bone tissue by fibrous connective tissue comprising varying amounts of mineralized substances [6]. FOLs are categorized in three main groups: cemento-osseous dysplasia (COD), fibrous dysplasia (FD), and cemento-ossifying fibroma (COF). Between them, COF is a neoplastic lesion. FOLs have similar histopathologic features; therefore, the diagnosis is based on patient history, clinical, and radiographic correlation [6–8]. COD, which is always seen in the tooth-bearing areas of the jaws, has three subtypes: periapical COD (PCOD), focal COD (FoCOD), and florid COD (FCOD). PCOD affects the periapical region of the mandibular incisors, and FoCOD represents the periapical lesions of the posterior mandible, while FCOD affects multiple segments of the jaws [7].

As COF is a neoplasm, in such cases, the lesion removal is the main treatment, and in all cases, the teeth involved in the lesion were also removed. Therefore, patients are seeking tooth replacement [9]. In two other lesions, dental implant placement in dysplastic tissue was encountered in patients with consequences such as dental implant failure [4].

The aim of this study is to provide an overview for clinicians to understand the long-term results of dental implants placed near or inside active/resected fibro-osseous lesions in human jaws, as well as any complications post-operation, and all reported management methods.

## Methods and materials

### Protocol and registration

This systematic review is written according to the Preferred Reporting Items for Systematic Reviews and Meta-Analyses guideline (PRISMA) [10], and its protocol is prospectively registered at PROSPERO (CRD420251270949).

### Eligibility criteria

The PICO question of the present study is as follows:

What are the failure rates and treatment plan options (O) of implant placement (I) in patients with a fibro-osseous lesion who need an implant treatment at the site of the lesion (P)?

No control group nor a comparator group has been determined for the question to maximize the number of retrieved results.

#### Inclusion criteria

Original research articles published in English regardless of their study design, were included in this systematic review having the following criteria:

- P: Patients with requiring and implant treatment at the site of a fibro-osseous lesion.
- I: Implant treatment has been performed for the patients.
- C: Studies can either comprise or not comprise any control group.
- O: Reported failure rate, success rate, or survival rate of the implants placed in the fibro-osseous lesions.

#### Exclusion criteria

- Articles that their full-texts could not be reached or retrieved.
- Studies that detail of lesions in its samples were not clearly determined.
- In vitro and in vivo investigations, review studies, preprints, and conference papers or abstracts.

### Information sources and search

An electronic search was performed in PubMed, Web of Science, and Scopus up to June 2026. The search was conducted using adapted keywords and Medical Subject Headings (MeSH). The following terms have been used for the search in the PubMed database:

((fibro-osseous lesions OR fibro osseous lesions OR fibrous dysplasia OR FD OR cemento-osseous dysplasia OR focal cemento-osseous dysplasia OR florid cemento-osseous dysplasia OR periapical cemento-osseous dysplasia OR FCOD OR FLCOD OR PCOD OR (Fibrous Dysplasia of Bone[MeSH Terms]) OR (cemento ossifying fibroma[MeSH Terms])) AND (dental implant OR dental implants))

Articles were selected for further evaluation. Furthermore, a manual search through the reference lists of the included articles was conducted to find any missing article.

### Study selection

Endnote 21 (Clarivate, Philadelphia, USA) was utilized for reference management. after elimination of all duplicates, all titles and abstracts were screened by two independent reviewers (FAM and AFA). Then, two researchers (FAM and SAM) independently evaluated the full-texts of included articles. In case of discrepancy occurrence at any step, it was resolved by a discussion with a fourth reviewer (AA).

### Data extractions

Two reviewers (FAM and AFA) extracted information from included articles independently. Afterward, a third reviewer (AA) read the collected data to check for discrepancies. Collected data items included authors name and publication year, participant characteristics (Age, sex, and any systemic conditions), characteristic, diagnosis, and location of the lesion followed by its treatment, study outcomes, and occurrence of any complication followed by its management, and follow up duration.

### Risk of bias assessment

Included studies were appraised by NIH tools for quality assessment. Based on the study designs, the appropriate risk of bias assessment tool was used. The quality of studies has been evaluated independently by two authors (FAM and SAM), and all disagreements were resolved by (AA).

## Results

### Search Results

**Fig. 1:**
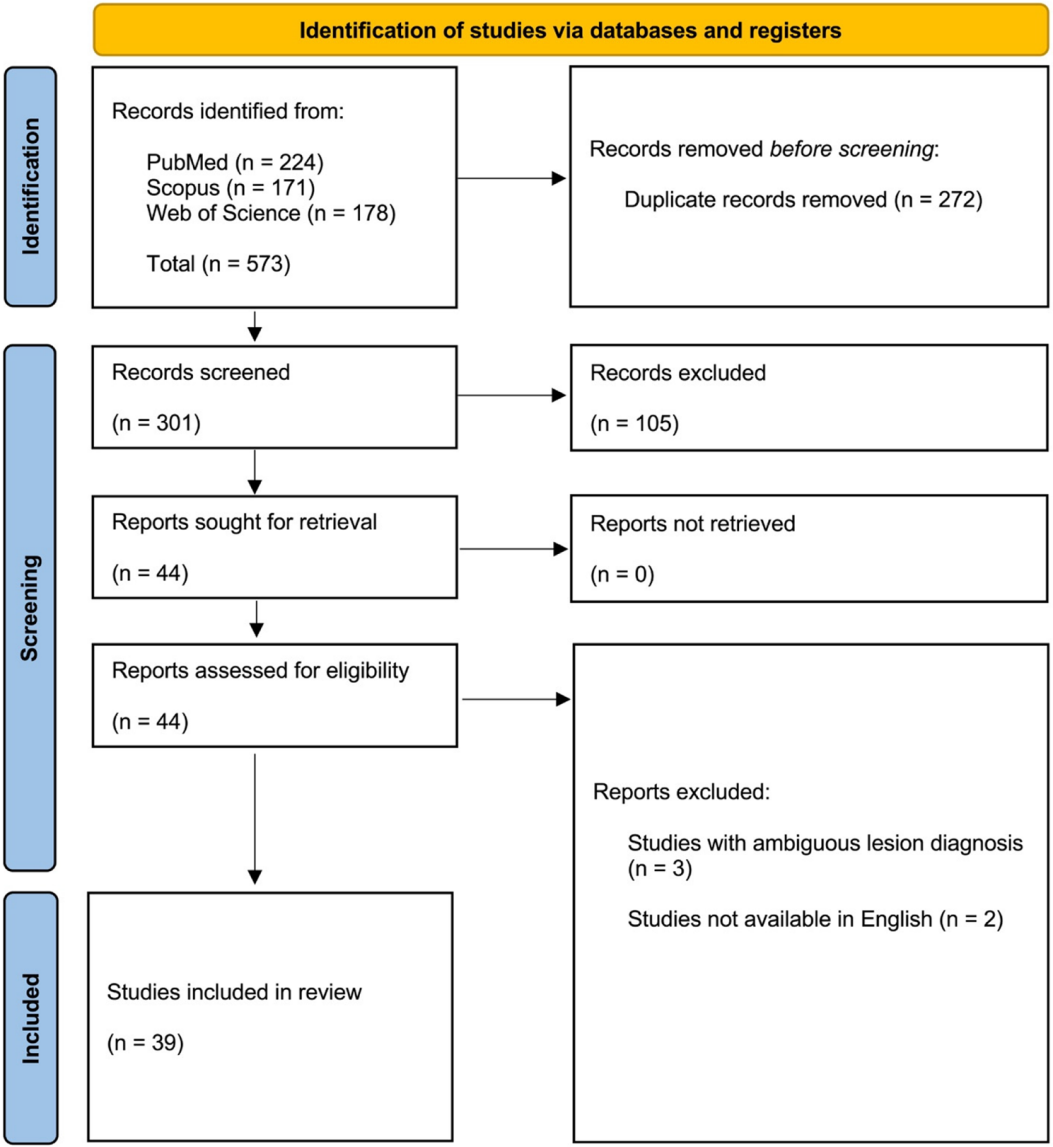
The PRISMA flow diagram of the study

Figure 1 depicts the PRISMA flowchart of this study. 301 studies were found during the electronic search through the databases and the reference list of the relevant articles. 44 articles have been selected for a thorough full-text screening after the appraisal of the titles and abstracts. This evaluation process culminated in a final inclusion of 39 eligible studies in the present systematic review.

### Study characteristics and qualitative synthesis

#### COD

Table 1 summarizes the findings of the included studies on the insertion of the dental implants into the COD lesions. Sixteen case report studies comprising 21 patients (19 females and two males) with ages ranging from 35 to 73, reported on the outcomes of placing implants in the COD lesions [5, 11–25]. Except for the Shaheen study [23] in which the lesion was in the canine and premolar area of the maxilla, all other lesions were in the mandible, two in the incisor area [15, 22], one in the canine to premolar area [23], and others in the posterior mandibular areas. In total, 40 implants were placed in the COD lesions, healthy areas near the lesions, or in the grafted tissues placed in the location of the removed lesions; nine of which have undergone implant failure (22.50% Failure rate). The follow-up period on the included studies ranged from three months to eight years. In one study [21], the authors removed the lesion from the posterior mandible and reconstructed it with a free fibula graft, and preferred to place the dental implants in the non-involved areas at the anterior mandible. In three of the included studies, the dental implant has been placed in the healthy bone near the lesion [5, 23, 25]. Two studies removed the lesions, placed a bone graft in the area, and placed the dental implants in the grafted tissue [19, 24]. All nine implant failures occurred for the implants placed in the dysplastic tissue. In 11 studies, all or some of implants were placed in the dysplastic tissue [11–18, 20, 22, 24], in seven of which [11–13, 17, 18, 20, 22] complications such as pus discharge, swelling, pain, peri-implantitis, implant failure, tenderness, and osteomyelitis [13, 17] have occurred. Management of these complications was described as the lesion and implant removal procedure [11–13, 17, 18, 20, 22]. One study reported a guided bone regeneration procedure following the implant removal and lesion debridement [18]. Two studies changed the treatment plan and decided to use a fixed prosthesis for the rehabilitation of the areas of the implant removal [20, 22].

**Table 1:** The summary of studies investigating the placement of implants in cemento-osseous dysplasia lesions

Study	Sex / Age / Systemic condition	Location / Clinical presentation	Treatment	Follow up / Complication	Treatment after complication / Follow up	Implants Placed in the lesion, in healed or grafted tissue, or in healthy bone near the lesion / Implants Failed
Sun et al., 2026 [11]	F / 47	COD lesion at the 1st mandibular molar region with the need for implant	-Implant inserted in the 1st mandibular molar region into the dysplastic tissue of the COD lesion -After four months, the provisional prosthesis was delivered for the implant	12 Mo / After 12 months of follow-up, the patient came back with the symptoms of the sensation of a loose implant with radiographic signs of a circumferential radiolucency around the implant	-Explanting the implant with enucleation and debridement of the peri-implant lesion -After nine months, the healing process was uneventful	1 / 1
Hu et al., 2025 [12]	6 Patients (5 F / 1 M) Age: 40–73 (Median: 45)	Posterior mandible	-Four implants were placed with no contact to the lesion in the healthy bone -Two implants were placed in partial contact with the lesion -Five implants were embedded in the lesions, in the dysplastic tissue -One implant was placed in the location of removed lesion on which was filled with autogenous graft composed of bone chips.	1–9 Y / Two implants placed within the dysplastic tissues failed after 2 Y of post-restoration due to peri-implantitis	-Maintenance and therapeutic approaches were applied to the two implants over the course of 1 Y post peri-implantitis -Removal of two implants	12 / 2
Graf et al., 2025 [24]	F / 54 -History of breast cancer treated with surgery and radiotherapy 2 Y earlier	1st mandibular molar / Continuous infection after tooth extraction (Purulent discharge and facial cellulitis) (Osteomyelitis)	-AB therapy/ No improvement -Local resection surgery + AB therapy -Autogenous and allogenic bone grafting -Digital guided implant placement, one in the healthy bone, one in the graft, and one in the dysplastic tissue (After 8 Mo) -Soft tissue graft to increase and reinforcement of keratinized tissue (After a few Mo) -Prosthesis delivery after 3 Mo	13 Mo / No		3 / 0
Kim et al., 2024 [13]	F / 60	Mandibular 2nd molar	-Dental implant (Placed 5 Y before in the site of dysplastic tissue)	5 Y / Presence of extra-orally fistula and pus discharge occurred few Mo after peri-implant gingivectomy (Osteomyelitis)	-Applying a dressing to the affected site + NSAID + AB therapy -Removing dental implant and inferior lesion -3 Mo follow up	1 / 1
Park et al., 2023 [25]	F / 42	Mandibular 1st molar/ Pain, chewing discomfort and mobility –Persistent even after RCT and apicoectomy (Lesion removal)	-Core-biopsy from distal root -Dental implant placement at the mesial root socket (Healthy bone) -Particulate bone substitute placement in the peri-implant defects covered with resorbable collagen membrane -Uncovering procedure after 6 Mo -Fixed prosthetic restoration 2 Mo later	1 Y / No		1 / 0
Mlouka et al., 2022 [14]	F / 48	Mandibular 2nd molar / Asking for Replacing missing teeth	-Accomplishing drilling sequence in dysplastic tissue + AB therapy (Pain and swelling resulted in diagnosis of abscess, drainage + betadine wash) -Placing dental implant 3 W after 1st surgery -Healing abutment placement after 3 Mo after	NA / NA		1 / 0
Perez et al., 2021 [15]	F / 45	Mandibular central incisors/ Asking for Replacing missing teeth due to periodontitis	-Two dental implants placement in dysplastic tissue -Marginal bone loss -Connective tissue grafting -Fixed prosthetic restoration after 7 Mo	6 Mo / No		2 / 0
Shadid and Kujan, 2020 [16]	F / 44	Mandibular 1st and 2nd molars / Asking for Replacing missing teeth	-AB prophylaxis + placing Two dental implants in dysplastic tissue -Uncovering implants after 5 Mo -Fixed prosthetic restoration 1 Mo after	8 Y / No		2 / 0
Shin et al., 2019 [17]	F / 70 / Oral bisphosphonate for 1 Y and hypertension	Mandibular 2nd premolar, 1st and 2nd molars region	-One implants placed in healthy tissue and two other placed in dysplastic tissue	NA / Swelling and pain (Osteomyelitis)	-One dental implant in dysplastic tissue was removed before -Removing the sequestrum and the other dental implant in dysplastic tissue -Bone remodeling after 9 Mo	3 / 2
Park et al., 2019 [18]	M / 39 / Smoker, occasionally consuming alcohol	Mandibular 1st molar and 2nd premolar	Two implants placed in dysplastic tissue	16 Y / Peri-implantitis (Gingival edema, and bleeding, pus discharge, and masticatory discomfort)	-Two dental implants were removed + debridement + GBR	2 / 2
Shaheen, 2019 [23]	F / 35	Maxillary canine to 2nd premolar / Asking for dental treatment	-Atraumatic tooth extraction -Two Implant placement in healthy bone (Canine and 2nd premolar area) near dysplastic tissue -Fixed prosthetic restoration 3 Mo	3 Mo / No		2 / 0
Esfahanizadeh and Yousefi, 2018 [5]	F / 62	Mandibular 1st and 2nd Molar/ Asking for Replacing missing teeth	-Two Implant placement in healthy bone near the lesion -Fixed prosthetic restoration after 6 Mo	1 Y / No		2 / 0
Sukegawa, 2016 [19]	F / 55	Persistent pain even after multiple endodontic therapy of left 1st mandibular molar (Osteomyelitis)	-AB therapy / No improvement -Removing the lesion and extracting tooth -Autogenous bone block graft from retromolar area -Dental implant placement in grafted tissue after 4 Mo -Provisional restoration -Definitive prosthesis	5 Y / No		1 / 0
Oliveira, 2014 [22]	F / 40	Mandibular central incisor	-Dental implant placement 6 Mo before	6 Mo / Dental implant spontaneously fallen out (Implant failure)	-Biopsy of the area (Uneventful healing after 1Y) -Tooth replacement with adhesive fixed prosthesis	1 / 0
Gerlach, 2013 [20]	F / 37	-Mandibular 1st molar / spontaneous pain, tenderness to percussion, and periapical radiolucency	-RCT of tooth due to periapical radiolucency around apex / No improvement (Misdiagnosis of COD with endodontic lesion) -Tooth extraction + bone graft -Dental implant placement + final restoration after 6 Mo	26 Mo / Implant mobility, swelling (Bone expansion), tenderness, pain on palpation	-Implant and lesion removal -Replacement of fixed partial prosthesis on adjacent teeth 1 Y follow up	1 / 1
Bencharit et al., 2003 [21]	F / 58	-Posterior mandibular region / Pain and swelling associated with complete denture for 10 Y (Diagnosis of COD and 4 surgeries were done) -Intra-oral fistula (Chronic osteomyelitis)	-Failure of conventional treatments for osteomyelitis -Partial mandibulectomy and reconstruction with free fibula graft -5 Implant placement in non-involved area (Anterior of mandible)	3 Y / No		5 / 0

*AB* Antibiotic, *F* Female, *M* Male, *Mo* Months, *Y* Years, *W* Weeks, *RCT* Root canal therapy, *GBR* Guided bone regeneration, *NA* Not available

#### FD

Table 2 summarizes the findings of the included studies on the insertion of the dental implants into the FD lesions. Fourteen studies consisting of 14 patients (13 female and one male) with ages ranging from 8 to 79, reported on the outcomes of placing implants in the FD lesions [4, 26–38]. Seven studies reported the FD lesion in the maxilla [4, 26, 28, 29, 31, 32, 38], six studies reported the lesion in the mandible [27, 30, 33–35, 37], and one study reported a case with polyostotic FD associated with McCune-Albright syndrome in both maxilla and mandible [36]. Fifty-six implants were placed in either of the FD defects, healed areas post removal of the FD lesions, or in the location of the removed FD lesions reconstructed with grafted tissues; and, from these placed dental implants, four implants failed (Two of them were placed in dysplastic tissue) with a 7.14% failure rate. The follow-up period on the included studies ranged from six months to 12 years. Regarding the radiographic findings, 11 studies have mentioned a “Ground-glass” [4, 26–30, 34–38] appearance, and only one study reported “a poorly defined mixed lesion of radiolucent and radiopaque areas” [33].

**Table 2:** The summary of studies investigating the placement of implants in fibrous dysplasia lesions

Study	Sex / Age	Location	Clinical presentation	Treatment	Follow up	Complication	Implants Placed in the lesion, in healed or grafted tissue, or in healthy bone near the lesion / Implants Failed
Kablan et al., 2025 [4]	F / 58	Right Maxilla	-Facial asymmetry	-Digital guide bone contouring using piezosurgry + digital guide four bone core were extracted by the trephine + GBR using bone substitute (Xenograft) -Four dental implants placed using digital guide in grafted tissue (After 5 Mo) -Final prosthesis (After 4 Mo)	NA	No	4 / 0
Chen et al., 2025 [27]	F / 32	Mandibular 1st molar	-Expansion of the alveolar crest on the edentulous site (Tooth extracted 3 Y ago) caused inadequate restorative space -Pseudo-pocket in the proximal of adjacent teeth	-Complete curettage of the lesion by piezoelectric osteotomy -Performing biopsy again to confirm the healing site (After 2 Y) -One dental implant placed in the healthy healed bone (4 Y after first osteotomy) -Final prosthesis (After 3 Mo)	1 Y	No	1 / 0
Amrou et al., 2024 [28]	F / 63	Maxillary molars and premolars	-Expansion of the alveolar crest on the edentulous site -The residual stumps of the posterior maxillary teeth appeared covered by the overgrowth of alveolar bone and gingiva	-Two dental implants were in dysplastic tissue (Unknown duration), one of them loaded -Osseous recontouring and prescribing antibiotics	6 Mo	No	2 / 0
Kablan, 2023 [26]	F / 35	Left Maxilla	-Mild facial asymmetry -Mild expansion of the vestibular left posterior maxillary ridge	-Removing the 1st molar + removing a box-shaped (2 cm × 1 cm × 2 cm) of dysplastic tissue from the maxillary ridge to the floor of maxillary sinus + particulate bone substitute (Xenograft) + Pedicled buccal fat pad graft for primary closure **-**Four dental implants placed in grafted tissue (After 6 Mo) -Implants uncovery (After 4 Mo) + Acrylic temporary bridge -Fixed ceramic prosthesis (After 1 Y)	7 Y	No	4 / 0
Lizio et al., 2023 [29]	F / 79	Right Maxilla	-Progressive slow growing painless hard swelling of the right maxilla (10 Y duration) -Facial asymmetry -Masticatory problems -Enlarged alveolar process and hemi- palate swelling	-Under general anesthesia bone recontouring + extraction of the teeth -Five dental implants placed (With surgical guide) in dysplastic tissue and two in normal tissue of another site in the maxilla -Final fixed prosthesis (after 6 Mo)	6 Y	Slowly growing vestibular bony swelling in right maxilla (During 2 Y) (Recurrence) -One dental implant had peri-implantitis lead to implant removal -Prosthesis reconnected to the other fixtures	5 / 1
Adnot et al., 2019 [30]	F / 64	Posterior mandible (FD diagnosed since childhood)	-Swelling -Decrease vertical dimension	-Bone recontouring + Two dental implants placed in dysplastic tissue + three dental implants placed in the other normal site -Final prosthesis (After 6 Mo)	2 Y	No	3 / 0
Carlo et al., 2019 [31]	F / 27	Right maxilla (FD diagnosed before)	-Swelling	-Maxillary reshaping + free fibula flap placement in 2001 -Four dental implants placement in the site of remodeled free fibula graft (After 2 Y) -Resin cement-retained temporary prosthesis (After 1 Y) -Peri-implant inflammation treated with free gingival graft (After 3Y)	12 Y	-Peri-implantitis of Two implants -Removal of two dental implants + GBR -Two dental implant placement in grafted site (After 6 Mo) -Final prosthesis (After 9 Mo)	4 / 2
Sosin et al., 2015 [32]	F / 29	Right Maxilla (FD diagnosed before with three times debulking procedures)	palato-alveolar defect after partial maxillectomy resulted in oronasal fistula	-Wide resection of the spongiform lesion + iliac free flap surgery -Debulking surgery (After 7 Mo) -Three dental implants placed in grafted tissue (After 3 Mo)	1.5 Y	No	3 / 0
Shnaiderman-Shapiro et al., 2015 [33]	F / 59	Posterior Mandible		-Three dental implants were in dysplastic tissue	NA	-Pain and swelling in the area of one dental implant (Osteomyelitis) -Removing the implant + curettage and removing the sequestrum + antibiotic therapy -The lesion was not resolved -Long-term treatment with doxycycline	3 / 1
Petrocelli and Kretschmer, 2014 [34]	F / 10	Mandible (Craniofacial FD case)	-Swelling -Tooth mobility	-Extraction of mobile teeth + recontouring of mandible -Bilateral recontouring of mandible (After 1 Y) -Removing the lesion from mandible symphysis + iliac crest graft (After 5 Y) -Six dental implants placed in grafted tissue + another iliac crest graft (After 6 Mo) -Implants uncovery + vestibuloplasty (After 8 Mo)	17 Mo	No	6 / 0
Monje et al., 2013 [38]	F / 49	Maxillary Posterior	-Asymptomatic slow growing bone expansion (4 Y duration) -Pain	-Extraction of involved teeth + curettage -Two dental implant placed in healed tissue + GBR with bone substitute material (Xenograft) (After 10 Mo) -Final prosthesis (After 6 Mo)	1 Y	No	2 / 0
Caridad and Platas, 2008 [35]	F / 35	Mandible	-Swelling, pain and paresthesia	-Removal of lesion + curettage -iliac crest graft immersing in PRP (After 2 W) -Five dental implants placed in grafted tissue (After 3 Mo) -Final prosthesis (After 2 Mo)	7 Y	No	5 / 0
Bajwa et al., 2008 [36]	F / 32	Maxilla and Mandible (Polyostotic FD) diagnosed for 13 Y (McCune-Albright syndrome with several debulking procedures)	-Facial asymmetry -Partial edentulous jaw with reduced inter arch space	-Contouring alveolar ridge (For adequate interocclusal clearance) + coronoidectomy (For mouth opening) + Nine dental implants placed in dysplastic tissue (5 in maxilla and 4 in mandible) (After 9 Mo) -Implants uncovery (After 5 Mo) -Two other dental implants placed immediately after tooth extraction -Final prosthesis (After 5 Mo)	5 Y	No	9 / 0
Chang et al., 2004 [37]	M / 8	Mandible	-Asymptomatic, growing bony enlargement (For few Mo) -Facial asymmetry -Midline deviation	-Bone trimming for 2 times (Interval of 5 Y) -Radical resection of mandible (After 3 Y) + fibula graft + Five dental implants placed in the fibula graft -Implants uncovery + FGG (After 1 Y) - Final prosthesis (After 1 Mo)	2 Y	No	5 / 0

*F* Female, *M* Male, *PRP* Platelet-rich plasma, *PFD* Polyostotic fibrous dysplasia, *Y* Years, *Mo* Months, *NA* Not available, *PA* Peri-apical, *CBCT* Cone beam computed tomography, *GBR* Guided bone regeneration

Two studies mentioned that they have removed the FD lesion and placed the dental implants in the healed healthy bone: one after 10 months [38] and another one after 4 years [27]. In seven of the included studies, the dental implant has been placed in the grafted tissue three months to two years post-lesion-removal [4, 26, 31, 32, 34, 35, 37]; from which, two studies used GBR technique with xenogeneic materials for grafting [4, 26], two studies used fibula grafts [31, 37], and three studies used iliac crest grafts [32, 34, 35]. In one of these studies [31], two implants placed in the fibula graft underwent failure due to peri-implantitis. Two studies reported a wide and radical resection of the structure for lesion removal [32, 37]. However, other studies mentioned more conservative removal methods such as recontouring and curettage. On the other hand, five studies placed their implants in the FD dysplastic lesions [28–30, 33, 36]; two of which reported implant failure due to peri-implantitis [29] and osteomyelitis [33]. Managing the complications, the studies reported implant removal and redesigning the prosthesis [29], post-implant-removal GBR and placing new dental implants [31], and lesion curettage plus sequestrum removal and adjunctive antibiotic therapy [33]. However, in the Shnaiderman-Shapiro et al. study, the management protocol was not successful for resolving the lesion, and the patient was finally prescribed a long-term doxycycline regimen [33].

#### COF

Table 3 summarizes the findings of the included studies on the insertion of the dental implants into the COF lesions. Nine studies with two male and seven female patients with ages ranging from 16 to 41, were available on the outcomes of placing implants in the COF lesions [9, 39–46]. Five studies reported a lesion at the posterior of the mandible [9, 41–44], three studies reported a lesion within the anterior and posterior of the mandible [40, 45, 46], and one study reported a lesion at the posterior of the maxilla [39]. Twenty-nine dental implants (Three of which failed [10.34% Failure rate]) were placed in the location of the removed COF lesions, reconstructed with grafted tissues or spontaneously healed bone tissue. The follow-up period on the included studies ranged from nine months to 10 years. Regarding the radiographic findings, five studies mentioned mixed radiolucent and radiopaque lesions [9, 39, 41, 42, 45] and four studies mentioned a radiolucent lesion [40, 43, 44, 46]. Five studies reported a well-defined lesion [40–42, 44, 45], of which three studies mentioned a sclerotic margin [40, 41, 44]. On the other hand, one study mentioned a partially ill-defined margin [9]. Root resorption and displacement of the mandibular canal were reported in three [9, 45, 46] and two [9, 45] studies, respectively.

**Table 3:** The summary of studies investigating the placement of implants in cemento-ossifying fibroma lesions

Study	Sex/Age/Systemic condition	Location / Clinical presentation	Treatment	Follow up of lesion removal / Recurrence	Follow up of dental implants/ Complication	Implants Placed in the healed or grafted tissue / Implants Failed
Shinozaki et al., 2025 [9]	M / 22	Posterior mandible / Painless, progressive bone expansion (1 Y duration)	-Segmental mandibulectomy involving teeth + Contouring mandible with a custom-made titanium mesh tray + Bridging IAN gap with a 4-mm m diameter PGA-collagen nerve conduit -Secondary augmentation using iliac cortical bone and PCBM graft (After 18 Mo) -Two dental implants placement (digital guided) in the grafted site (After 12 Mo) -Final prosthesis (After 6 Mo)	6.5 Y / No	42 Mo / No	2 / 0
Bedi et al., 2022 [39]	F / 14	Posterior maxilla / Non-tender bone swelling (2 Mo duration)	-Lesion enucleation + Extraction of involved teeth + PRG -Interim removable prosthesis -Three dental implants placement in healed site (After 4 Y) -Final Hybrid prosthesis (After 6 Mo)	4.5 Y / No	NA / NA	3 / 0
Lamartine De Moraes Melo Neto et al., 2019 [40]	F / 41	Anterior and posterior mandible / Tooth mobility and displacement	-Lesion enucleation + Extraction of involved teeth + Reconstructing with tibial bone graft + Titanium screen -Three dental implant placement in grafted tissue (After 19 Mo) -Final prosthesis	5 Y / No	41 Mo / No	3 / 0
Rodrigues Arantes et al., 2019 [41]	M / 20	Posterior mandible / Tooth mobility and displacement, asymptomatic bone swelling	-Partial mandibulectomy (Preserving part of ascending ramus) + Contouring mandible with titanium plate and bicortical screws -Secondary augmentation using iliac cortical bone + allogeneic graft + titanium mesh + PRP + undifferentiated mesenchymal cells (After 12 Mo) -Three dental implants placement in grafted tissue (After 16 Mo) - Final prosthesis (After 8 Mo)	36 Mo / No	NA/ NA	3 / 0
Yoshimura, 2013 [42]	F / 35	Posterior mandible/ Asymptomatic swelling (5 Y duration), Lingual inclination of 2nd molar	-Segmental mandibular resection + iliac and greater auricular nerve (due to sacrificing inferior alveolar nerve) graft + titanium plate -Surgical removing the titanium plate (After 13 Mo) -Two Dental implants placement in grafted tissue (After 7 Mo) -Final prosthesis (After 6 Mo)	10 Y / No	94 Mo / No /	2 / 0
Blanco and Ostrosky, 2013 [43]	F / 16	Posterior mandible / No clinical symptom	-Mandibular resection + reconstruction with free fibula flap -Interposing spongy iliac crest bone graft between two slices of fibula graft and fixing with miniplates (After 4 Mo) -Five dental implants placement (After 24 Mo) -Final prosthesis	40 Mo/ No	12 Mo / No (a mesial dental implant was not placed in the proper position and not restored in the final prosthesis	5 / 0
Pal et al., 2012 [44]	F/ NA	Posterior mandible / Slowly progressive bone swelling	-Conservative excision of lesion + extraction of involved teeth + bone fill using xenograft bone substitute -Two dental implant placement in grafted tissue (After 6 Mo) - Final prosthesis (After 3 Mo)	9 Mo / No	NA / NA	2 / 0
Gurol et al., 2001 [45]	F / 17	Anterior and posterior of mandible / Asymptomatic bone swelling	-Conservative excision of the lesion + extraction involved teeth -Orthodontic treatment and orthognathic surgery for jaw discrepancies -Six implant placed (After 38 Mo) in healed site (Three dental implants with simultaneous bone graft) - Overdenture placement (After 5 Mo)	43 Mo / No	NA / Three dental implants were failed as a result of bone graft exposure (2 Mo after placement)	6 / 3
Abdulwassie and Dhanrajani, 2001 [46]	F / 22	Anterior and posterior of mandible / Bone swelling, transient tingling sensation of the lower lip	-Complete enucleation and curettage of lesion + extraction of involved teeth -Three dental implants placement healed surgical site (After 2 Y) -Final prosthesis (After 5 Mo)	41 Mo / No	1 Y / No	3 / 0

*F* Female, *M* Male, *Mo* Months, *PGA* Poly-glycolic acid, *PCBM* Particulate cancellous bone and marrow, *PRG* Platelet-rich gel, *PRP* Platelet-rich plasma, *Y* Years

In five studies, the authors decided to conduct enucleation and curettage of the lesion; three of which have placed the implants at the sites healed without grafting [39, 45, 46], and the other two have used tibial bone graft (Lamartine De Moraes Melo Neto et al., 2019) and xenogeneic bone graft [44]. However, Bedi et al. have used platelet-rich gel at the time of enucleation, and Gurol et al. utilized the GBR procedure simultaneously with the placement of three of their implants. On the other hand, four studies performed mandibular resection for the lesion removal [9, 41–43]. Shinozaki et al. [9] performed a segmental mandibulectomy, contoured the mandible with titanium mesh, and grafted the inferior alveolar nerve (IAN) with 4 mm of PGA-collagen scaffold. After 18 months, at the second step of augmentation, they have used two iliac cortical plates filled with particulate cancellous bone and marrow. Twelve months later, the implants were placed, and six months after that, the prostheses were delivered. Rodrigues Arantes et al. [41] conducted a partial mandibulectomy with the preservation of the ascending ramus and contoured the mandible with titanium mesh. After 12 months, they have used titanium mesh, iliac cortical plates, allogenic bone grafts, platelet-rich plasma, and undifferentiated mesenchymal stem cells for the augmentation. Sixteen months later, the implants were placed, and eight months after that, the prostheses were delivered. In the Yoshimura study [42], segmental mandibular resection with bone grafting using titanium plates and iliac bone grafts, and nerve grafting using the greater auricular nerve were performed. After 13 months, the plates were removed. The dental implants were placed and loaded seven and 13 months after the plate removal, respectively. Blanco and Ostrosky [43] conducted a mandibular resection combined with a reconstruction process using a free fibula flap. Spongy iliac crest bone grafts were interposed between the slices of fibula grafts and fixed with miniplates four months after the first surgery. Twenty-four months later, dental implants were inserted into the grafted tissue.

Regarding the complications, only Gurol et al. [45] reported the failure of three of their implants, on which a simultaneous GBR procedure had been performed, and the implants failed as a result of the graft exposure after two months post placement.

### Risk of Bias Assessment

The summary of the included studies’ quality has been provided in Table 4 (COD), Table 5 (FD), and Table 6 (COF). All domains except Q7 demonstrated a low risk of bias in the included studies. The Q7 domain representing the adequacy of the follow-up periods and four studies on placing implant in the COD lesions [14, 15, 17, 23], three studies on placing implants in the FD lesions [4, 28, 33], and one study on placing implant in the COF lesions [44] had a high risk of bias in this domain; and, as a result all included studies received a “Good” rating at the quality assessment, except the aforementioned articles which have received a “Fair” rating,.

**Table 4:** The Summary of the risk of bias assessment of studies reported on the placement of dental implants in the cemento-osseous dysplasia lesions

Authors	Q1	Q2	Q3	Q4	Q5	Q6	Q7	Q8	Q9	Quality Rating
Sun et al., 2026 [11]	√	√	√	--	√	√	√	---	√	Good
Hu et al., 2025 [12]	√	√	√	--	√	√	√	---	√	Good
Graf et al., 2025 [24]	√	√	√	--	√	√	√	---	√	Good
Kim et al., 2024 [13]	√	√	√	--	√	√	√	---	√	Good
Park et al., 2023 [25]	√	√	√	--	√	√	√	---	√	Good
Mlouka et al., 2022 [14]	√	√	√	--	√	√	×	---	√	Fair
Perez et al., 2021 [15]	√	√	√	--	√	√	×	---	√	Fair
Shadid and Kujan 2020 [16]	√	√	√	--	√	√	√	---	√	Good
Shin et al., 2019 [17]	√	√	√	--	√	√	×	---	√	Fair
Park et al., 2019 [18]	√	√	√	--	√	√	√	---	√	Good
Shaheen, 2019 [23]	√	√	√	--	√	√	×	---	√	Fair
Esfahanizadeh and Yousefi, 2018 [5]	√	√	√	--	√	√	√	---	√	Good
Sukegawa, 2016 [19]	√	√	√	--	√	√	√	---	√	Good
Oliveira, 2014 [22]	√	√	√	--	√	√	√	---	√	Good
Gerlach, 2013 [20]	√	√	√	--	√	√	√	---	√	Good
Bencharit et al., 2003 [21]	√	√	√	--	√	√	√	---	√	Good

1. Was the study question or objective clearly stated?

2. Was the study population clearly and fully described, including a case definition?

3. Were the cases consecutive?

4. Were the subjects comparable?

5. Was the intervention clearly described?

6. Were the outcome measures clearly defined, valid, reliable, and implemented consistently across all study participants?

7. Was the length of follow-up adequate?

8. Were the statistical methods well-described?

9. Were the results well-described?

**Table 5:** The Summary of the risk of bias assessment of studies reported on the placement of dental implants in the fibrous dysplasia lesions

Authors	Q1	Q2	Q3	Q4	Q5	Q6	Q7	Q8	Q9	Quality Rating
Kablan et al., 2025 [4]	√	√	√	--	√	√	×	---	√	Fair
Chen et al., 2025 [27]	√	√	√	--	√	√	√	---	√	Good
Amrou., et al. 2024 [28]	√	√	√	--	√	√	×	---	√	Fair
Kablan, 2023 [26]	√	√	√	--	√	√	√	---	√	Good
Lizio et al., 2023 [29]	√	√	√	--	√	√	√	---	√	Good
Adnot et al., 2019 [30]	√	√	√	--	√	√	√	---	√	Good
Carlo et al., 2019 [31]	√	√	√	--	√	√	√	---	√	Good
Sosin et al., 2015 [32]	√	√	√	--	√	√	√	---	√	Good
Shnaiderman-Shapiro et al., 2015 [33]	√	√	√	--	√	√	×	---	√	Fair
Petrocelli and Kretschmer, 2014 [34]	√	√	√	--	√	√	√	---	√	Good
Monje et al., 2013 [38]	√	√	√	--	√	√	√	---	√	Good
Caridad and Platas, 2008 [35]	√	√	√	--	√	√	√	---	√	Good
Bajwa et al., 2008 [36]	√	√	√	--	√	√	√	---	√	Good
Chang et al., 2004 [37]	√	√	√	--	√	√	√	---	√	Good

1. Was the study question or objective clearly stated?

2. Was the study population clearly and fully described, including a case definition?

3. Were the cases consecutive?

4. Were the subjects comparable?

5. Was the intervention clearly described?

6. Were the outcome measures clearly defined, valid, reliable, and implemented consistently across all study participants?

7. Was the length of follow-up adequate?

8. Were the statistical methods well-described?

9. Were the results well-described?

**Table 6:** The Summary of the risk of bias assessment of studies reported on the placement of dental implants in the cemento-ossifying fibroma lesions

Authors	Q1	Q2	Q3	Q4	Q5	Q6	Q7	Q8	Q9	Quality Rating
Shinozaki et al., 2025 [9]	√	√	√	--	√	√	√	---	√	Good
Bedi et al., 2022 [39]	√	√	√	--	√	√	√	---	√	Good
Lamartine De Moraes Melo Neto et al., 2019 [40]	√	√	√	--	√	√	√	---	√	Good
Rodrigues Arantes et al., 2019 [41]	√	√	√	--	√	√	√	---	√	Good
Yoshimura, 2013 [42]	√	√	√	--	√	√	√	---	√	Good
Blanco and Ostrosky, 2013 [43]	√	√	√	--	√	√	√	---	√	Good
Pal et al., 2012 [44]	√	√	√	--	√	√	×	---	√	Fair
Gurol et al., 2001 [45]	√	√	√	--	√	√	√	---	√	Good
Abdulwassie and Dhanrajani, 2001 [46]	√	√	√	--	√	√	√	---	√	Good

1. Was the study question or objective clearly stated?

2. Was the study population clearly and fully described, including a case definition?

3. Were the cases consecutive?

4. Were the subjects comparable?

5. Was the intervention clearly described?

6. Were the outcome measures clearly defined, valid, reliable, and implemented consistently across all study participants?

7. Was the length of follow-up adequate?

8. Were the statistical methods well-described?

9. Were the results well-described?

## Discussion

This systematic review was designed and executed to gather all data regarding the long-term results of dental implants placed near or inside active/resected fibro-osseous lesions in human jaws, as well as any complications post-operation, and all reported management methods.

FOLs are a heterogeneous group of pathologies in which normal bone is replaced by fibrous tissues containing varying amounts of mineralized material [47–50]. COD is a dysplastic/reactive lesion that occurs almost exclusively in tooth-bearing sites of the jaws. There are three main subgroups for COD: PCOD, FoCOD, and FCOD [6, 47]. FD is a developmental non-neoplastic bone disorder caused by GNAS mutations. FD has three main subtypes: monostotic FD, polyostotic FD, and craniofacial FD [50, 51]. COF is a benign neoplastic fibro-osseous lesion with three subtypes: conventional, juvenile trabecular, and juvenile psammomatoid [48, 50, 52].

Dental implant placement in patients with FOLs in the jaws remains a controversial treatment choice since these lesions are characterized by varying degrees of abnormal bone remodeling, altered vascularity, sclerosis, and impaired healing potential. Historically, fibro-osseous lesions were considered relative or even absolute contraindications for dental implantations. However, increasing numbers of clinical reports and review papers have demonstrated that implant therapy might still be feasible in selected cases with precise diagnosis, surgical planning, and post-operative maintenance [12, 53–55].

The concept of implant failure must first be clarified before discussing implant therapy and the management of its complications in fibro-osseous lesions. Implant failure can be categorized into either biological or mechanical/prosthetic failures. Albrektsson et al. defined a successful dental implant as one that is immobile, free of any peri-implant radiolucency, and experiences less than 1.5 mm of marginal bone loss and less than 0.2 mm annually, after the initial first year of insertion [56]. Implant failure might include lack of osseointegration, progressive peri-implant bone loss, implant mobility, chronic peri-implantitis, persistent pain, suppuration, or complete implant loss [56–58]. Implant failures can be divided into early and late failures; early failures occur before prosthetic/functional loading and are commonly associated with impaired osseointegration caused by surgical trauma, overheating, infection, poor primary stability, or inadequate healing [59]. Late implant failures happen after functional loading and are generally correlated with biomechanical overload, peri-implantitis, systemic disease, smoking, or compromised bone quality [60]. In healthy bone structures, systematic reviews have reported excellent 10-year survival rates exceeding 94% [61, 62].

### COD

A total of 40 dental implants were inserted inside or near COD lesions, with 9 of them eventually failing; all were inserted inside affected/dysplastic bone. The overall rate of dental implant failure was at 22.50%. In the current systematic review, 16 case reports with a total of 40 dental implants placed in the jaws of COD patients were included; 9 of these implants reportedly failed. In 9 of these studies, dental implants were placed inside the dysplastic tissue, which resulted in complications reported in 5 of these studies and consequential implant removals.

COD lesions are usually centered around the alveolar process and periodontal ligament (PDL), making it a crucial bone condition in dental implantology and periodontology [6, 47]. As COD lesions mature, healthy bone is replaced by fibrous tissues, progressive calcification occurs, vascularity decreases, and dense sclerotic masses start to develop. All of this will result in a hypo-vascular bone with poor/limited healing capabilities [47, 63]. PCOD localizes around apices of anterior mandible teeth, usually has limited expansion, and its early radiolucency progresses to mixed/radio-opaque lesions. In PCOD, teeth usually remain vital; however, it frequently mistaken for endodontic diseases, which often leads to unnecessary root canal treatments. Dental implants might fail in mature sclerotic areas of PCOD lesions due to compromised vascularity [6, 47]. FoCOD is a solitary lesion that often affects the posterior mandible with the possibility of mild expansion. FoCOD usually has no effect on teeth; however, it can occasionally be associated with the alveolar regions of recently extracted teeth. In FoCOD lesions, localized sclerosis may reduce healing and increase the risks of secondary infection after dental implantation [47, 64]. In FCOD, there are multifocal involvements of multiple quadrants, dense lobulated sclerotic masses, markedly reduced vascularity, and predisposition to chronic osteomyelitis. The FCOD subtype, out of all the COD subtypes, has the greatest clinical significance and risks for oral surgery and implantology. FCOD can have serious effects on teeth (i.e., secondary periodontal compromise, tooth mobility (in advanced FCODs), difficult extractions due to dense bone, and increased risk of post-extraction infection). FCOD is generally considered a moderate-to-absolute contraindication for dental implants due to compromised osseointegration, poor bone turnover, chronic osteomyelitis triggered by surgical trauma, and elevated implant infection risk [47, 63, 64]. Similar to FD, resection of COD-affected bone solely for the goal of creating a grafted implant site is generally not the preferred strategy; unnecessary surgeries within the dysplastic lesion should be avoided, and implant positioning should be planned in a way to minimize or avoid direct contact with COD lesions.

Mature Stage III COD lesions, which contain denser mineralized tissues, have much better implant survival than Stage II lesions. Mature mineralized tissues (i.e., Stage III) may provide improved mechanical stability for implant anchorage compared to immature fibro-cellular lesions (i.e., Stage II). On the other hand, the dense sclerotic architecture of mature lesions may simultaneously compromise vascularity and immune responses, leading to potentially predisposing patients to delayed infection and osteomyelitis. Therefore, lesion maturation (i.e., higher stages) may have both beneficial and detrimental biological effects.

Amongst all fibro-osseous lesions, COD is the most frequently reported lesion associated with implant placement, particularly the FCOD and FoCOD. As COD lesions mature, they become increasingly avascular and sclerotic, predisposing patients to poor healing and secondary infection; this hypo-vascularization is particularly important since osseointegration heavily depends on adequate blood supply and bone remodeling [65, 66]. Despite these concerns, recent evidence suggests that implant placement is not universally contraindicated in COD cases.

### FD

There was a total of 56 dental implants inserted in or near FD lesions, 4 of which failed and were extracted; 2 of the failed implants were inserted inside the dysplastic affected bone, and 2 of them were inserted inside the healed bone reconstructed with bone graft/particles. The overall rate of dental implant failures inserted near or inside FD-affected bone was at 7.14% across all 14 patients. In FD lesions, normal cancellous bone gets replaced by fibro-cellular tissue and immature woven bone, producing poorly organized and mechanically weaker bone [50, 51]. FD can have various negative impacts on alveolar bone: diffused expansion of maxilla or mandible, “ground-glass” radiographic appearance, cortical thinning, altered trabecular architecture, and reduced normal marrow spaces. The maxilla is more commonly affected by FD compared to the mandible, and the lesions may cross sutures into adjacent craniofacial bone structures [51]. FD can lead to tooth displacement, delayed eruption, malocclusion, root divergence, rare root resorption, and occlusal plane distortion. However, in FD lesions, despite the displacement, teeth usually remain vital [51, 64]. Dental implant insertion in FD lesions is highly controversial due to the abnormal bone remodeling, unpredictable primary stability, altered implant positioning over time (caused by ongoing lesion activity), and heterogeneous bone density. That being said, some investigations have reported successful osseointegration in quiescent, mature lesions throughout long-term evaluation periods. On the contrary, risks of implant failure in active or expanding lesions are considerably higher [47, 51, 64]. Regarding dental implantation and prosthetic options for affected jaw bones with FD, most clinicians still prefer implant placement in stable/healed FD bone rather than radical resection [26]. GBR in FD cases is promising but still suffers from weak evidence in the literature; in Kablan et al.’s case report, stable function of a dental implant 7 years after its placement inside a grafted portion of the FD-affected bone was reported [26]. Guided implant surgery is highly valuable in FD cases; guided surgery helps maximize engagement of native healthy bone, avoid vital structures, and improve prosthetically driven positioning [67, 68]. In summary, the current evidence in the literature suggests the following order of choices for FD treatment plans: (1) implants anchored in healthy native bone, using CBCT-guided surgical planning; (2) partial or full placement of implants inside quiescent FD-affected bone; (3) limited resection/osteotomy of dysplastic alveolar bone with GBR or particulate graft reconstruction followed by delayed implant placement.

Overall, the literature suggests that with careful surgical planning and correct placement of implants near the FD lesions or within the resected/healed bone, low failure rates can be expected. In 2019, Adnot et al. reported a case in which dental implants were inserted in both left and right posterior mandible with edentulism; however, the left side was treated after the resection of FD. Two years post-operation, both sides had desirable osseointegration and no complications were reported [30].

### COF

Across the 9 included case reports, there was a total of 29 implants inserted in the healthy bone around the previous COF lesion or inside the healed/reconstructed affected bone with 3 of them reportedly failing; all 3 failed implants were inserted inside healed/grafted bone and were from one patient who had 6 initial implants placed in their jaw (10.34% Failure rate). COF is a true benign neoplasm with well-circumscribed growth and centrifugal expansion [48, 50, 52]. COF is an expansile lesion with well-defined borders, which causes cortical thinning, bowing, and mandibular predilection (in the premolar/molar region). Unlike FD, COF lesions are usually sharply demarcated from surrounding bone [48, 52]. Similar to FD, COF can cause tooth displacement, root divergence and resorption, and occlusal disturbance. Large lesions caused by COF may significantly distort alveolar anatomy [48]. In regard to management/treatment of COF lesions, enucleation/resection often leads to major bony defects; dental implants are usually delayed until complete healing and confirmation of no recurrence. Reconstructed bone-grafted sites may later be suitable to receive dental implants; however, long-term monitoring is crucial since recurrence is common, particularly in younger patients [48, 52]. Juvenile form of COF is a way more aggressive type, which affects the maxilla and mandible; it causes rapid expansion, cortical destruction, with the possibility of maxillary sinus and orbital extension. Most importantly, it has a high recurrence rate. Similar to FD and COF, it can lead to marked tooth displacement, failed eruptions, root resorption, and severe malocclusion. During the active phase of juvenile COF, dental implants are usually contraindicated owing to their aggressive lesions, substantial recurrence rates, extensive surgical resections, and the limiting factor of ongoing skeletal growth in younger patients. Hence, implant placement gets postponed until confirmed disease stability [48, 50].

As discussed before, the literature consistently indicates that COFs are expansile neoplasms with ongoing bone remodeling and unpredictable mineralization [69–71]. Clinically, COF might be asymptomatic and only discovered accidentally on radiographies. COF can also lead to significant esthetic and functional changes. In some cases, it can be challenging to distinguish between COF and FD owing to considerable radiologic and histologic overlaps [72]. One of the key features of COF is its relatively considerable recurrence rate; a systematic review by MacDonald-Jankowski reported recurrence rates of up to 12% [73], which is aligned with other reports in the literature [69, 71, 74]. Nine studies with a total of 29 placed implants were available on the outcomes of implant insertion in patients with COF lesions, in which only three implants failed due to the GBR graft exposure. Several studies with reported successful implant-based rehabilitation in COF cases suggest that enucleation, curettage, bone reconstruction (i.e., grafting and guided bone regeneration), and delayed implant placement can all help the final outcome [41, 44, 46]. Considering the unique nature of COF, peri-implant bone may remodel unpredictably, implant support can be lost, and surveillance imaging becomes more challenging; hence, most clinicians prefer delayed implant placement after complete lesion removal, and long-term radiographic follow-up before functional/prosthetic loading. More importantly, the bone quality inside COF, even in healed/passive cases, might be inadequate for predictable osseointegration. COF contains cellular fibrous stroma, variable calcified materials, immature woven bone, and cementum-like calcifications; hence, this tissue cannot act like normal mature alveolar bone. Consequently, drilling and implant insertion in and around these poorly structured affected bones can lead to poor drilling feedback, irregular osteotomy preparation, inadequate torque value, overheating risk, and inconsistent bone density. It is highly suggested that the following precautions be considered for implant placement in COF-affected bone and surrounding area: careful sequential drilling, copious irrigation, under-preparation if necessary, and achieving high primary stability [75, 76].

### Radiographic stages and clinical implications of FOLs

COD lesions typically progress through three stages: (1) osteolytic stage (completely radio-lucent); (2) mixed/cementoblastic stage (radio-lucent lesion with internal radio-opaque deposits); and (3) mature stage (predominantly radio-opaque lesion, often with a radiolucent rim) [77, 78]. Early periapical radiolucency might mimic inflammatory endodontic lesions, particularly when associated with loss of lamina dura; however, involved teeth are generally vital [79]. The early radiolucent stage of COD is frequently mistaken for periapical granuloma or radicular cyst due to its appearance at the tooth apices. Early FD lesions may appear radiolucent; with maturation they develop a mixed appearance and eventually the characteristic ground-glass radio-opacity [80]. COF radiographically evolves from its radiolucent stage to mixed radiolucent-radiopaque stage to predominantly radiopaque stage; COF lesions remain well circumscribed and sharply demarcated throughout development, helping distinguish them from FD [81, 82].

The key clinical difference between COF with FD and COD is the fact that COF behaves as a localized tumor, whereas FD and COD are disorders of bone formation and remodeling. In COF lesions, the pathologic tissue itself is the problem; therefore, treatment aims to remove the lesion. Since the tumor is usually circumscribed, excision is feasible and often definitive [50]. On the other hand, in FD and COD lesions, immediate removal of the lesion is not the main focus of the treatment. In FD, the lesion is integrated into the skeleton via a mosaic genetic defect; there is no clear boundary to remove, hence, treatment focuses on function and deformity rather than eradication of diseased tissue [83]. COD lesions are not biologically aggressive and often remain stable for years. Since surgery can compromise already hypo vascular bone, the preferred management is usually avoidance of unnecessary intervention [53, 84].

### Study limitations and suggestions

- All included studies in the current systematic review and almost all available reports in the literature are case reports. The lack of any meaningful human clinical trial, randomized or not, has led to significant uncertainties regarding the indication/contraindication and the risks of dental implants in relation to varied fibro-osseous lesions.
- Future researchers are highly encouraged to design and execute clinical trials, or if only case reports/series are feasible, it is highly suggested that split-mouth dental implantation be considered; one side with the affected bone and one side with healthy native bone.
- The calculated pooled failure ratio reported is not site-specific (regarding the position of implant related to the lesion). Furthermore, the failure ratio reported is a result of simply pooling the cases together from different case-reports and ignores patient-level clustering. Hence, special caution should be made when interpretating the failure rates and adopting them for clinical decision making.
- A lot of studies had a vague description of the success/failure of inserted dental implants.
- The evaluation periods were varied amongst studies. Future researchers are encouraged to follow up on the health of the implants and their surrounding bones for at least the first 12 months post-surgery.
- Some studies have reported that these fibro-osseous lesions were “missed” before or even during the insertion of dental implants. To prevent these diagnostic mistakes, it is recommended that clinicians take all radiographic (i.e., CBCT) and pathologic precautions in times of uncertainty.
- Regarding the two investigated dysplasia, COD and FD, clinicians and researchers are highly encouraged to explain to their patients that since replacing their teeth that are inside or around these lesions would be so challenging, they must be really careful in trying to protect their teeth and prevent dental extraction and dental implantation. Moreover, in the events of tooth extraction and dental implantation, careful considerations must be taken with strict evaluation periods over the years.

## Conclusion

Considering the limitations of the present study, it can be concluded that placing dental implants embedded in the fibro-osseous lesions can be associated with a higher failure rate. It would be more appropriate to avoid placing dental implants into the COD and FD lesions and inserting the implants in the surrounding healthy bone, healed, or grafted bone tissues are preferred. However, due to the neoplastic nature of COF removing the lesions prior to the implant insertion and using grafting and delayed implant insertion and loading methods to achieve a higher survival rate is crucial.

## Data Availability

No datasets were generated or analysed during the current study.

## Abbreviations

COD: Cemento-osseous Dysplasia
COF: Cemento-ossifying Fibroma
FCOD: Florid Cemento-osseous Dysplasia
FD: Fibrous Dysplasia
FoCOD: Focal Cemento-osseous Dysplasia
FOL: Fibro-osseous Lesion
PCOD: Periapical Cemento-osseous Dysplasia
